# Time-course gene expression data on the transcriptional effects of Aminaphtone on ECV304 endothelial cells

**DOI:** 10.1016/j.dib.2016.06.051

**Published:** 2016-07-02

**Authors:** Giulia Salazar, Chiara Bellocchi, Katia Todoerti, Federica Saporiti, Luca Piacentini, Raffaella Scorza, Gualtiero I. Colombo

**Affiliations:** a Referral Centre for Systemic Autoimmune Diseases, University of Milan and Fondazione IRCCS Ca׳ Granda Ospedale Maggiore Policlinico, Milano, Italy; b Laboratory of Preclinical and Translational Research, IRCCS-CROB, Referral Cancer Centre of Basilicata, Rionero in Vulture, Italy; c Laboratory of Immunology and Functional Genomics, Centro Cardiologico Monzino IRCCS, Milano, Italy

**Keywords:** Aminaphtone (PubChem CID: 84621), Endothelial cells, Transcriptome, Inflammation, Vasoactive drug

## Abstract

We previously showed that Aminaphtone, a drug used in the treatment of chronic venous insufficiency, modulates several vasoactive factors, such as endothelin-1 and adhesion molecules. Here, we provide data of time-course experiments about the effects of Aminaphtone on gene expression at the genome-wide level in human endothelial cells undergoing cytokine stimulation *in vitro*. ECV-304 endothelial cells were incubated with interleukin-1β (IL-1β) in the presence or absence of Aminaphtone for 1, 3, and 6 h. Gene expression profiles were analyzed by microarray. This article contains complete data on the genes significantly modulated by the drug over time. The data are supplemental to our original research article reporting detailed analysis of the actions of Aminaphtone on IL-1β stimulated endothelial cells at the molecular level, "Gene expression profiling reveals novel protective effects of Aminaphtone on ECV304 endothelial cells" (Salazar et al., 2016) [1].

**Specifications Table**

**Table:** 

Subject area	*Biology*
More specific subject area	*Cellular transcriptomics*
Type of data	*Tables, figure*
How data was acquired	*Affymetrix 7G Microarray Scanner (Affymetrix, Santa Clara, CA)*
Data format	*Filtered, analyzed*
Experimental factors	*Time-course experiments of gene expression responses of confluent human ECV304 endothelial cells, stimulated with recombinant IL-1β (100 IU/ml), to treatment with Aminaphtone (6 μg/ml) vs. medium alone*
Experimental features	*Whole-genome gene expression analysis performed at 1, 3, and 6-h time points using GeneChip Gene 1.0 ST Arrays (Affymetrix, Santa Clara, CA)*
Data source location	*Milan, Italy*
Data accessibility	*Filtered, analyzed data are reported within this article. Raw and normalized data are available via NCBI׳s GEO accession number GEO: GSE83297 (**http://www.ncbi.nlm.nih.gov/geo/query/acc.cgi?acc=GSE83297**).*

## Value of the data

• Previously unreported transcriptional effects of Aminaphtone, a drug currently used for the treatment of chronic venous insufficiency.
• Illustrate gene expression changes induced by Aminaphtone in a human endothelial cell line subjected to inflammatory stimulus.
• May facilitate further experiments to unveil the still unknown mechanism of action of this drug.
• May serve as a benchmark for comparison with data obtained in primary cells for further insight.
• May stimulate further research on the clinical use of this drug in other disease conditions, in which inflammation and endothelial dysfunction are key pathophysiological elements.

## 1 Data

**Fig. 1:**
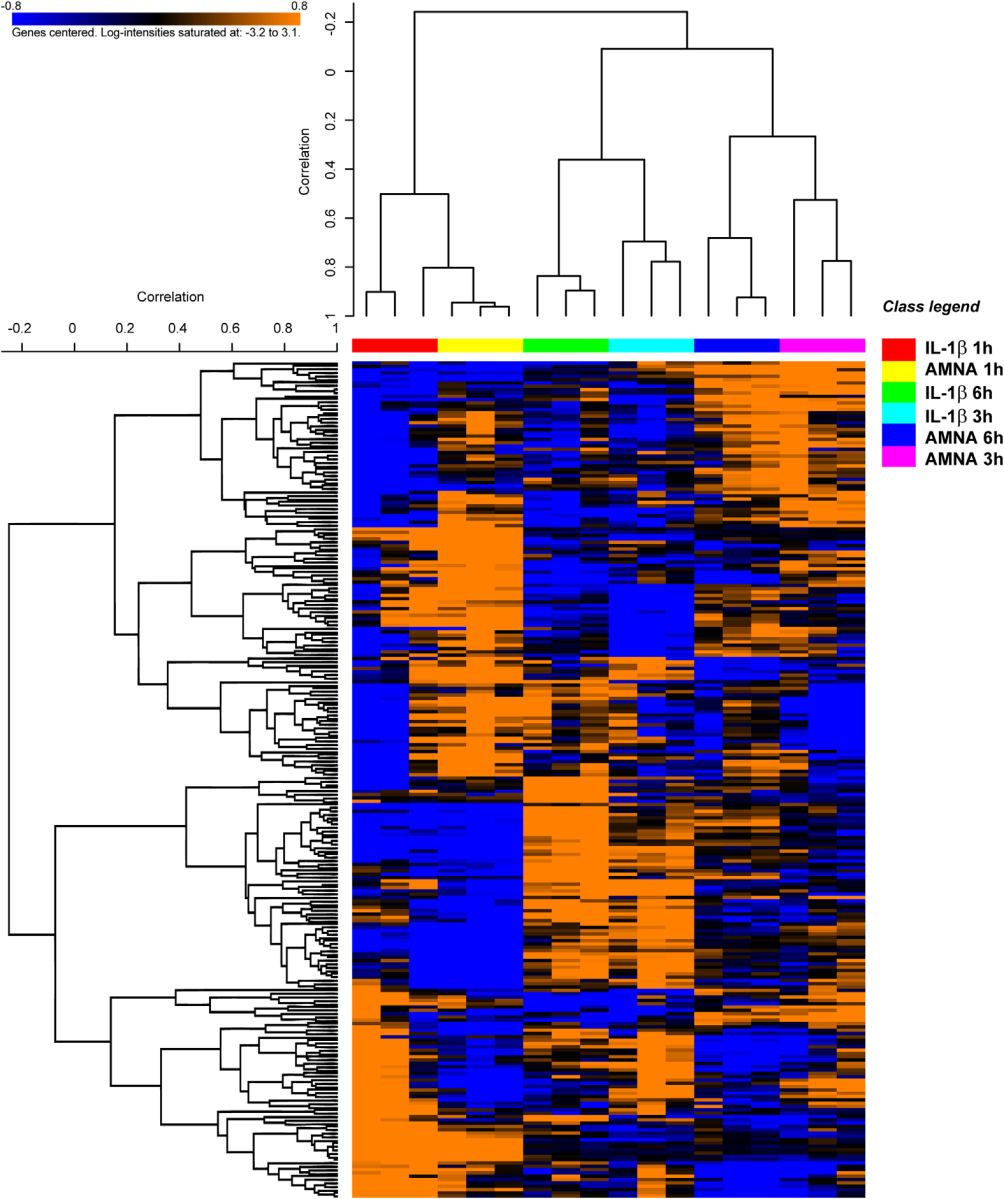
Unsupervised hierarchical clustering of significant genes modulated over time by Aminaphtone in treated *vs.* untreated IL-1β stimulated ECV304 endothelial cells. The BETR algorithm identified 252 significantly modulated genes (FDR <0.1). Samples and genes were clustered using Pearson׳s correlation (centered) and average linkage method. Each combination of treatments and time points regrouped in distinct clusters. The log_2_ transformed, normalized, median-centered expression level of each gene is represented with a blue, black, and orange color scale: blue indicates below median, black, equal to, and orange above median. Class legends stand for: IL-1β (IL-1β without Aminaphtone); AMNA (IL-1β with Aminaphtone).

Transcriptional effects of Aminaphtone treatment on human ECV304 endothelial cells stimulated with IL-1β for 1, 3 and 6 h are shown in Table 1. The complete raw and normalized data are deposited in NCBI׳s Gene Expression Omnibus [2] and are accessible through GEO Series accession number GEO: GSE83297 (http://www.ncbi.nlm.nih.gov/geo/query/acc.cgi?acc=GSE83297). A hierarchically clustered heatmap of the differentially expressed genes (Fig. 1) visualizes the arrangement of treatment groups into clusters. Table 2 illustrates data on the functional enrichment analysis of modulated genes, showing gene sets and molecular pathways affected by Aminaphtone in IL-1β stimulated ECV304 cells.

## 2 Experimental design, materials and methods

### 2.1 Cell cultures and treatments

Human ECV304 endothelial cells (European Collection of Authenticated Cell Cultures, ECACC No. 92091712) were seeded at ×10^5^/well in 6-well tissue-culture treated plates and grown to confluence in M199 complete medium (Invitrogen, Carlsbad, CA) supplemented with 10% fetal bovine serum (FBS; Hyclone, Logan, UT), at 37 °C in a humidified incubator with 5% CO_2_. Cells were serum starved (1% FBS) to synchronize the mitotic phase 24 h before treatment. Cells were then incubated for 1, 3, and 6 h with recombinant IL-1β 100 IU/ml (Sigma-Aldrich, St. Louis, MO) in the presence of Aminaphtone 6 μg/ml (Baldacci, Pisa, Italy) or an equal volume of medium alone. Experiments were performed in three independent replicates for each time point.

### 2.2 RNA isolation and whole-genome gene expression profiling

Total RNA extraction was performed with TRIzol (Life Technologies, Rockville, MD) directly added to the ECV304 culture plates. Removal of contaminating genomic DNA was done by treating RNA samples with RNase-free Turbo DNase (Life Technologies) for 15 min at room temperature. RNA quantity and quality were assessed respectively by micro-volume spectrophotometry on an Infinite 200 PRO plate reader (Tecan, Männedorf, Switzerland) and by on-chip capillary electrophoresis on a Bioanalyzer 2100 (Agilent Technologies, Santa Clara, CA). Absorbance ratio at 260 and 280 nm was ≥1.9 and the RNA integrity number (RIN) was >8 for all samples.

For each replicate, 100 ng of total RNA were amplified and labeled using the Whole-Transcript Sense Target Labeling Protocol by Affymetrix (Santa Clara, CA) without ribosomal RNA reduction. Affymetrix GeneChip Human Gene 1.0 ST arrays were hybridized with 11 µg of labeled sense DNA, washed, stained, and scanned on an Affymetrix 7G Scanner according to the manufacturer׳s protocols.

### 2.3 Data processing and probe mapping

Data were extracted using the Affymetrix Expression Console software. Background correction, log_2_ transformation, quantile normalization, and median polish probeset summarization was performed using the Robust Multi-array Average (RMA) method [3] implemented in the RMAExpress software v1.0.5. The resulting dataset consisted of 28869 probesets. The BRB-ArrayTools v4.3.2 (developed by Dr. Richard Simon and BRB-ArrayTools Development Team) and Bioconductor *R* packages v2.12 [4] were used for probe filtering and annotation. Probesets were deemed as non-informative and excluded from further analysis under any of the following conditions: *P*-value of the log-ratio variation greater than 0.01, *i.e.* genes showing minimal variation across samples; 17th percentile of intensity less than 10, *i.e.* genes with the lowest acceptable expression level at most in three samples. Multiple probesets were reduced to one per gene symbol by using the most variable probe measured by interquartile range across arrays. After applying these stringent quality control and gene filtering criteria, we analyzed the expression changes over time of 6461 genes. Project was annotated with the Bioconductor annotation package hugene10sttranscriptcluster.db v8.0.1.

### 2.4 Statistical and bioinformatics analysis

Differentially expressed genes were sought combining two statistics implemented in the software MultiExperiment Viewer (MeV) v4.9 [5]. To identify genes varying significantly between the two conditions across time points, we used the Bayesian Estimation of Temporal Regulation (BETR) method [6], which is a linear random-effect modeling framework that takes into account correlations within samples between sampling times. Genes assigned a False Discovery Rate (FDR) <0.1 were deemed significantly modulated. Then, to determine which genes were mainly influenced by the effect of Aminaphtone treatment *per se* (*i.e.* irrespective of the time response) and/or by the interaction effect of the two factors time and treatment, we applied a two-factor ANOVA to the gene list identified by BETR, given the balanced factorial design of the study. Genes were considered statistically significant if the *P*-values either for treatment and/or for interaction were <0.05. We finally performed post-hoc pairwise comparisons (2-tailed Student׳s *t*-test) to identify significant differences (*P*<0.05) between treatment classes at any time points.

Unsupervised hierarchical clustering was performed using the algorithms implemented in BRB-ArrayTools, to visualize similarities and differences in gene expression profiles that could discriminate treatment classes and/or changes over time. Log_2_ transformed, normalized gene expression values were median-centered, scaled, and clustered by Pearson׳s centered correlation and average linkage as distance metrics.

Functional analysis of significant genes identified by BETR was carried out by examining gene sets for differential expression between Aminaphtone treated and untreated samples. Gene sets were derived from the Gene Ontology (GO) database (http://www.geneontology.org) [7], the Kyoto Encyclopedia of Genes and Genomes (KEGG; http://www.genome.jp/kegg/pathway.html), and the curated gene sets of the Broad Institute Molecular Signature Database (MSigDB) [8]. LS/KS permutation tests were used to find gene sets with more genes differentially expressed among the phenotype classes than expected by chance. The threshold *P*-value was set at 0.005. Redundant GO terms were filtered out using the web-based tool REViGO [9], allowing a similarity threshold of 0.5.

## Figures and Tables

**Table 1:** Differentially expressed genes in Aminaphtone-treated *vs.* untreated IL-1β-stimulated ECV304 endothelial cells.

**Symbol**	**Gene name**	**Gene ID**	**FDR**	***P***_***treatment***_	***P***_***interaction***_	**FC AMNA/IL-1β**			***Pairwise***		
						**FC 1 h**	**FC 3 h**	**FC 6 h**	**1 h**	**3 h**	**6 h**
***AAK1***	AP2 associated kinase 1	22848	9.13e-02	**5.98e-03**	7.26e-01	1.55	1.66	1.31		*	
***ACAP1***	ArfGAP with coiled-coil, ankyrin repeat and PH domains 1	9744	1.65e-02	**2.07e-04**	4.01e-01	−1.50	−1.23	−1.36	**		*
***AGAP2***	ArfGAP with GTPase domain, ankyrin repeat and PH domain 2	116986	1.73e-03	**8.86e-05**	4.53e-01	−1.39	−1.58	−1.31	**	**	*
***AK4***	adenylate kinase 4	205	8.79e-03	4.27e-01	**7.56e-04**	1.41	−1.01	−1.64	*		**
***ANKRD1***	ankyrin repeat domain 1 (cardiac muscle)	27063	8.02e-03	**2.75e-02**	**1.23e-02**	1.26	−1.46	−1.78		*	**
***ANKRD13A***	ankyrin repeat domain 13A	88455	8.98e-02	**2.37e-04**	1.88e-01	1.23	1.47	1.18	*	**	
***ANXA3***	annexin A3	306	4.39e-04	7.76e-02	**7.05e-04**	1.48	−1.33	−1.70	**	*	**
***AREG***	amphiregulin	374	7.88e-02	**6.01e-03**	6.32e-01	−1.37	−1.39	−1.78			*
***ARHGEF26***	Rho guanine nucleotide exchange factor (GEF) 26	26084	7.10e-02	**1.36e-03**	9.35e-01	1.42	1.33	1.42	*		*
***ARL4C***	ADP-ribosylation factor-like 4C	10123	2.31e-02	**2.22e-04**	8.41e-01	−1.41	−1.30	−1.39	**		**
***ATF7IP2***	activating transcription factor 7 interacting protein 2	80063	5.87e-02	**5.59e-03**	1.69e-01	1.77	1.17	1.22	**		
***BCL2L11***	BCL2-like 11 (apoptosis facilitator)	10018	8.59e-04	**7.06e-05**	**3.23e-02**	1.60	1.48	1.09	**	**	*
***BCL3***	B-cell CLL/lymphoma 3	602	4.12e-03	**3.18e-02**	**5.00e-03**	−1.98	−1.05	1.13	**		
***BMP6***	bone morphogenetic protein 6	654	1.78e-02	**3.25e-03**	6.12e-02	−1.00	1.65	1.47		**	*
***BNIP3***	BCL2/adenovirus E1B 19 kDa interacting protein 3	664	0.00e+00	**3.64e-05**	**4.09e-06**	1.11	−1.12	−2.54			**
***BRCA1***	breast cancer 1, early onset	672	7.46e-02	4.42e-01	**1.64e-02**	1.96	−1.28	−1.15	**		
***BUB1***	BUB1 mitotic checkpoint serine/threonine kinase	699	1.40e-02	5.76e-01	**5.38e-03**	1.93	−1.28	−1.26	**		
***BUB1B***	BUB1 mitotic checkpoint serine/threonine kinase B	701	5.11e-02	4.12e-01	**1.69e-02**	2.09	−1.32	−1.14	**		
***C14orf105***	chromosome 14 open reading frame 105	55195	6.13e-02	**4.75e-03**	5.28e-01	−1.22	−1.59	−1.62		*	*
***C15orf48***	chromosome 15 open reading frame 48	84419	5.83e-03	**7.16e-05**	**4.51e-02**	−1.61	−1.17	−1.25	**		*
***C1orf63***	chromosome 1 open reading frame 63	57035	3.07e-03	**1.82e-04**	3.43e-01	1.61	1.27	1.38	**		*
***C2orf44***	chromosome 2 open reading frame 44	80304	9.87e-03	**1.63e-03**	2.02e-01	1.79	1.35	1.20	**		
***C3orf58***	chromosome 3 open reading frame 58	205428	1.08e-05	3.22e-01	**1.35e-06**	1.54	1.17	−1.61	**	*	**
***C7orf53***	chromosome 7 open reading frame 53	286006	6.37e-05	**1.06e-03**	**1.05e-02**	2.19	1.05	1.27	**		
***C9orf131***	chromosome 9 open reading frame 131	138724	9.82e-03	**4.39e-04**	7.67e-02	1.67	1.21	1.19	**		
***CACHD1***	cache domain containing 1	57685	4.66e-02	**2.02e-03**	4.84e-01	1.31	1.63	1.29		**	
***CBLB***	Cbl proto-oncogene, E3 ubiquitin protein ligase B	868	8.71e-02	**1.29e-02**	2.19e-01	1.07	1.86	1.45		*	
***CCL22***	chemokine (C–C motif) ligand 22	6367	0.00e+00	**6.98e-06**	6.53e-02	−1.36	−2.20	−1.99	*	**	**
***CD70***	CD70 molecule	970	9.39e-02	**1.09e-03**	2.37e-01	−1.53	−1.22	−1.20	**		
***CD83***	CD83 molecule	9308	1.63e-03	**4.10e-04**	8.35e-02	1.80	1.22	1.27	**		
***CEBPD***	CCAAT/enhancer binding protein (C/EBP), delta	1052	6.06e-03	4.58e-01	**6.34e-04**	−1.70	1.12	1.31	**		*
***CENPF***	centromere protein F, 350/400 kDa	1063	8.68e-02	6.92e-01	**2.45e-02**	1.90	−1.75	−1.31	*		
***CEP55***	centrosomal protein 55 kDa	55165	4.42e-02	7.14e-01	**8.10e-03**	1.68	−1.40	−1.35	*		
***CEP76***	centrosomal protein 76 kDa	79959	7.67e-02	**2.29e-03**	1.52e-01	1.57	1.32	1.09	**	*	
***CHCHD7***	coiled-coil-helix-coiled-coil-helix domain containing 7	79145	6.62e-02	**3.19e-03**	4.05e-01	1.58	1.47	1.17	*	*	
***CITED2***	Cbp/p300-interacting transactivator, with Glu/Asp-rich carboxy-terminal domain, 2	10370	7.93e-02	2.38e-01	**3.69e-03**	−1.38	1.47	1.22	*	**	
***CKAP2L***	cytoskeleton associated protein 2-like	150468	1.53e-02	1.45e-01	**1.89e-02**	1.57	−1.97	−1.57		*	
***CLEC4E***	C-type lectin domain family 4, member E	26253	4.03e-03	**8.42e-04**	3.66e-01	−1.25	−1.53	−1.71		*	**
***CNNM4***	cyclin M4	26504	1.79e-07	**9.46e-05**	**4.77e-04**	−1.11	2.00	1.57		**	**
***CSF2***	colony stimulating factor 2 (granulocyte-macrophage)	1437	1.02e-03	**8.79e-04**	7.21e-01	−1.68	−1.88	−1.48	*	**	
***CTH***	cystathionase (cystathionine gamma-lyase)	1491	6.66e-03	**1.90e-04**	3.46e-01	1.29	1.59	1.31	*	**	*
***CTSK***	cathepsin K	1513	2.91e-03	**4.28e-04**	9.47e-01	1.54	1.53	1.45	*	*	*
***CUZD1***	CUB and zona pellucida-like domains 1	50624	7.05e-02	**3.72e-02**	**4.52e-02**	1.95	1.02	1.05	**		
***CX3CL1***	chemokine (C-X3-C motif) ligand 1	6376	1.25e-02	**3.11e-03**	4.58e-01	−1.94	−1.48	−1.33	**		
***CXCL3***	chemokine (C-X-C motif) ligand 3	2921	8.34e-02	**1.65e-02**	5.71e-02	−1.70	−1.34	1.07	**		
***CXCL6***	chemokine (C-X-C motif) ligand 6	6372	2.85e-03	**1.69e-04**	6.11e-01	−1.39	−1.34	−1.58	*	*	**
***CYB5D1***	cytochrome b5 domain containing 1	124637	2.40e-03	**1.21e-03**	7.39e-02	1.78	1.50	1.07	**	*	
***CYP24A1***	cytochrome P450, family 24, subfamily A, polypeptide 1	1591	2.62e-05	**4.54e-05**	7.89e-01	−1.52	−1.69	−1.51	**	**	**
***DBR1***	debranching enzyme homolog 1 (*Saccharomyces cerevisiae*)	51163	7.97e-02	**1.06e-03**	4.21e-01	1.52	1.26	1.24	**		
***DDIT4***	DNA-damage-inducible transcript 4	54541	5.57e-04	**5.87e-04**	5.05e-01	−1.90	−1.40	−1.57	**		*
***DDR1***	discoidin domain receptor tyrosine kinase 1	780	1.43e-02	**1.48e-03**	8.30e-02	−1.73	−1.26	−1.13	**		
***DENND2C***	DENN/MADD domain containing 2C	163259	3.14e-02	**1.33e-03**	9.57e-01	1.44	1.49	1.40	*	*	*
***DEPDC1***	DEP domain containing 1	55635	5.44e-02	9.05e-01	**1.02e-02**	1.75	−1.26	−1.44	*		
***DKK1***	dickkopf 1 homolog (*Xenopus laevis*)	22943	2.94e-05	**5.83e-05**	**1.71e-02**	−1.25	−1.25	−1.94			**
***DLEU1***	deleted in lymphocytic leukemia 1 (non-protein coding)	10301	7.23e-02	**5.02e-03**	1.27e-01	1.71	1.19	1.14	**		
***DLGAP5***	discs, large (Drosophila) homolog-associated protein 5	9787	4.29e-03	1.58e-01	**2.80e-03**	1.51	−1.58	−1.42	*	*	*
***DNAJB1***	DnaJ (Hsp40) homolog, subfamily B, member 1	3337	1.36e-02	**4.99e-03**	**4.35e-02**	1.82	1.31	1.00	**		
***DUSP8***	dual specificity phosphatase 8	1850	5.16e-03	**1.05e-02**	**4.26e-03**	−1.87	1.06	−1.07	**		
***EBI3***	Epstein-Barr virus induced 3	10148	0.00e+00	**6.30e-06**	6.87e-01	−1.82	−1.73	−2.06	**	**	**
***ECT2***	epithelial cell transforming sequence 2 oncogene	1894	6.84e-03	5.43e-02	**1.07e-02**	1.36	−1.84	−1.45		**	
***EDN1***	endothelin 1	1906	0.00e+00	**2.33e-06**	8.60e-01	−1.79	−1.93	−1.97	**	**	**
***EFNA1***	ephrin-A1	1942	4.91e-03	**1.20e-03**	**2.03e-02**	−1.74	−1.02	−1.31	**		*
***EFNB2***	ephrin-B2	1948	1.76e-02	**3.27e-03**	1.81e-01	1.81	1.40	1.13	**		
***EGR2***	early growth response 2	1959	7.52e-03	**3.57e-03**	**6.43e-03**	−1.80	−1.04	−1.06	**		
***EIF4B***	eukaryotic translation initiation factor 4B	1975	3.63e-02	4.03e-01	**9.48e-03**	1.56	−1.22	−1.69	*		*
***EIF4EBP2***	eukaryotic translation initiation factor 4E binding protein 2	1979	3.09e-04	**5.53e-06**	1.55e-01	1.25	1.56	1.42	*	**	**
***ELF3***	E74-like factor 3 (ets domain transcription factor, epithelial-specific )	1999	6.05e-02	**3.62e-02**	**8.45e-03**	−1.24	1.43	1.43		*	*
***ELMSAN1***	ELM2 and Myb/SANT-like domain containing 1	91748	3.96e-05	**1.34e-02**	**1.00e-05**	−1.86	1.19	1.08	**	*	
***ELOVL4***	ELOVL fatty acid elongase 4	6785	8.64e-03	**5.29e-04**	3.98e-01	1.40	1.62	1.25	*	**	
***ELOVL6***	ELOVL fatty acid elongase 6	79071	7.35e-02	1.86e-01	**1.29e-02**	1.84	−1.08	−1.15	**		
***ENO2***	enolase 2 (gamma, neuronal)	2026	6.53e-05	**1.50e-03**	**1.22e-02**	−1.27	−1.06	−2.31			**
***ERCC6L***	excision repair cross-complementing rodent repair deficiency, complementation group 6-like	54821	6.72e-02	8.17e-01	**1.39e-02**	1.75	−1.58	−1.21	*		
***ERF***	Ets2 repressor factor	2077	1.91e-02	**4.84e-04**	1.05e-01	−1.62	−1.22	−1.19	**		
***EVA1A***	eva-1 homolog A (*Caenorhabditis elegans*)	84141	1.25e-06	**3.14e-07**	**4.49e-03**	−1.19	−1.47	−1.74	*	**	**
***FAM102A***	family with sequence similarity 102, member A	399665	3.29e-02	6.69e-02	**5.48e-03**	−1.32	1.41	1.48		*	*
***FAM111B***	family with sequence similarity 111, member B	374393	7.05e-02	5.89e-01	**2.31e-02**	2.18	−1.58	−1.06	*		
***FAM46C***	family with sequence similarity 46, member C	54855	5.99e-04	**5.82e-04**	**4.90e-03**	1.87	1.20	1.03	**		
***FAM72D***	family with sequence similarity 72, member D	728833	2.54e-04	4.65e-01	**1.25e-03**	1.86	−1.60	−1.46	**	*	*
***FBLIM1***	filamin binding LIM protein 1	54751	1.21e-03	**1.31e-04**	5.98e-01	−1.61	−1.38	−1.39	**	*	*
***FBXL20***	F-box and leucine-rich repeat protein 20	84961	1.87e-04	**2.46e-04**	6.47e-02	1.19	1.91	1.40		**	*
***FBXO33***	F-box protein 33	254170	4.21e-02	**9.72e-04**	7.70e-02	1.22	1.62	1.13		**	
***FLJ36840***	uncharacterized LOC645524	645524	2.12e-02	1.52e-01	**2.64e-02**	2.66	−1.34	1.09	**		
***FOS***	FBJ murine osteosarcoma viral oncogene homolog	2353	4.65e-06	**4.26e-04**	**3.84e-04**	2.13	1.01	1.08	**		
***FRMD6***	FERM domain containing 6	122786	4.02e-02	**1.16e-02**	**3.29e-02**	1.11	−1.49	−1.57		*	**
***FRMD8***	FERM domain containing 8	83786	3.02e-02	**6.15e-03**	**2.25e-02**	−1.72	−1.23	1.03	**		
***FST***	follistatin	10468	7.49e-02	**1.12e-03**	1.27e-01	−1.07	−1.44	−1.42		**	**
***FUT11***	fucosyltransferase 11 (alpha (1,3) fucosyltransferase)	170384	5.23e-02	**1.79e-03**	**6.02e-03**	−1.14	1.00	−1.62			**
***GAN***	gigaxonin	8139	4.82e-02	**6.57e-03**	1.16e-01	1.80	1.22	1.12	**		
***GAREM***	GRB2 associated, regulator of MAPK1	64762	9.44e-02	**1.61e-03**	**1.88e-02**	−1.00	1.54	1.23		**	*
***GAS2L3***	growth arrest-specific 2 like 3	283431	5.77e-02	2.78e-01	**1.30e-02**	1.92	−1.14	−1.17	**		
***GCLC***	glutamate-cysteine ligase, catalytic subunit	2729	3.29e-02	**2.08e-03**	7.58e-01	1.56	1.53	1.32	*	*	
***GCLM***	glutamate–cysteine ligase, modifier subunit	2730	1.37e-02	**1.51e-03**	5.69e-01	1.71	1.40	1.35	**		
***GLCCI1***	glucocorticoid induced transcript 1	113263	2.69e-02	**1.03e-04**	8.16e-01	1.37	1.38	1.28	**	**	
***GPR56***	G protein-coupled receptor 56	9289	1.49e-02	**3.94e-03**	7.47e-02	−1.85	−1.09	−1.27	**		
***GPR89A***	G protein-coupled receptor 89A	653519	5.78e-02	**3.12e-03**	3.52e-01	1.67	1.32	1.22	**		
***GRB7***	growth factor receptor-bound protein 7	2886	1.09e-02	**1.07e-03**	5.88e-02	−1.74	−1.18	−1.18	**		
***GTF2B***	general transcription factor IIB	2959	6.15e-04	**8.75e-05**	1.99e-01	1.69	1.35	1.29	**	*	*
***HCAR1***	hydroxycarboxylic acid receptor 1	27198	9.99e-02	**3.02e-03**	7.95e-01	−1.29	−1.45	−1.49		*	*
***HILPDA***	hypoxia inducible lipid droplet-associated	29923	7.25e-05	**9.73e-04**	**3.21e-03**	−1.20	−1.01	−2.09			**
***HIST1H2AK***	histone cluster 1, H2ak	8330	3.09e-03	**2.95e-03**	1.11e-01	−1.05	−1.71	−1.85		*	**
***HIST2H2BF***	histone cluster 2, H2bf	440689	4.80e-03	**4.43e-05**	6.62e-01	1.36	1.32	1.47	**	*	**
***HIST2H4A***	histone cluster 2, H4a	8370	0.00e+00	**4.91e-07**	**1.85e-02**	1.87	1.46	1.32	**	**	**
***HK2***	hexokinase 2	3099	1.25e-06	**4.59e-04**	**1.65e-03**	1.06	−1.37	−2.25		*	**
***HMMR***	hyaluronan-mediated motility receptor (RHAMM)	3161	1.23e-02	2.21e-01	**8.49e-03**	1.58	−1.43	−1.71	*		*
***HMOX1***	heme oxygenase (decycling) 1	3162	1.69e-02	**3.20e-04**	3.02e-01	−1.51	−1.19	−1.41	**		**
***ID2***	inhibitor of DNA binding 2, dominant negative helix-loop-helix protein	3398	5.45e-03	**1.51e-04**	**1.15e-02**	1.38	1.58	1.03	**	**	
***IDI1***	isopentenyl-diphosphate delta isomerase 1	3422	6.89e-02	**4.10e-03**	2.47e-01	1.70	1.21	1.23	**		
***IGFL1***	IGF-like family member 1	374918	0.00e+00	**1.06e-05**	8.05e-01	−2.11	−2.05	−2.41	**	**	**
***IL1B***	interleukin 1, beta	3553	2.12e-03	**1.50e-03**	3.88e-01	−1.29	−1.92	−1.69		**	*
***IL32***	interleukin 32	9235	3.56e-03	**8.70e-05**	1.94e-01	−1.23	−1.34	−1.59		**	**
***IL7R***	interleukin 7 receptor	3575	2.09e-04	**7.60e-04**	8.60e-02	−1.11	−1.95	−1.69		**	**
***IRF2BP2***	interferon regulatory factor 2 binding protein 2	359948	1.26e-02	7.17e-02	**2.00e-04**	−1.65	1.11	1.13	**		
***ISCA1***	iron–sulfur cluster assembly 1 homolog (*S. cerevisiae*)	81689	2.42e-02	**2.41e-03**	1.97e-01	1.60	1.54	1.10	**	*	
***ISG20L2***	interferon stimulated exonuclease gene 20 kDa-like 2	81875	8.45e-02	**4.01e-02**	**2.40e-03**	−1.62	1.08	1.03	**		
***KCTD11***	potassium channel tetramerisation domain containing 11	147040	4.26e-05	**1.92e-04**	3.55e-01	−1.91	−1.36	−1.65	**		**
***KDELC1***	KDEL (Lys-Asp-Glu-Leu) containing 1	79070	5.23e-02	**7.58e-05**	3.32e-01	1.20	1.43	1.30		**	**
***KHDRBS3***	KH domain containing, RNA binding, signal transduction associated 3	10656	1.79e-03	**2.61e-06**	**7.74e-04**	1.05	1.57	1.32		**	**
***KIAA0922***	KIAA0922	23240	1.60e-02	**1.12e-03**	3.47e-01	1.25	1.68	1.35		**	
***KIAA1524***	KIAA1524	57650	2.09e-02	3.06e-01	**1.10e-02**	1.64	−1.68	−1.44	*	*	
***KIF14***	kinesin family member 14	9928	1.42e-02	8.55e-01	**1.29e-02**	2.46	−1.70	−1.31	*		
***KIF23***	kinesin family member 23	9493	7.31e-02	1.00e+00	**1.64e-02**	1.88	−1.46	−1.29	*		
***KLRAP1***	killer cell lectin-like receptor subfamily A pseudogene 1	10748	9.99e-02	9.35e-01	**2.08e-02**	1.86	−1.58	−1.22	*		
***KRT16***	keratin 16	3868	8.89e-04	**7.06e-04**	5.60e-01	−1.73	−1.76	−1.37	*	**	
***KRT17***	keratin 17	3872	1.35e-03	**6.95e-04**	2.03e-01	−1.86	−1.24	−1.43	**		*
***KRT34***	keratin 34	3885	6.82e-02	**5.18e-04**	8.08e-01	−1.27	−1.37	−1.40			**
***LAMB3***	laminin, beta 3	3914	9.31e-03	**7.05e-04**	8.37e-01	−1.57	−1.44	−1.39	**	*	*
***LIF***	leukemia inhibitory factor	3976	1.04e-03	**1.87e-02**	**1.93e-03**	−1.34	1.51	1.62	*	**	**
***LMCD1***	LIM and cysteine-rich domains 1	29995	7.13e-02	**2.87e-04**	8.53e-01	−1.38	−1.33	−1.28	**		
***LOC100130713***	uncharacterized LOC100130713	100130713	8.73e-03	**3.78e-02**	**1.06e-03**	−1.33	1.51	1.34	*	**	*
***LOC440896***	uncharacterized LOC440896	440896	1.04e-02	**7.89e-04**	8.40e-01	−1.55	−1.49	−1.37	*	*	
***LOX***	lysyl oxidase	4015	2.52e-02	**2.34e-04**	**3.94e-02**	−1.15	−1.19	−1.60			**
***LPIN1***	lipin 1	23175	1.40e-03	**2.40e-04**	**4.71e-02**	1.72	1.37	1.12	**	*	
***MARCH8***	membrane-associated ring finger (C3HC4) 8, E3 ubiquitin protein ligase	220972	7.20e-02	**2.41e-03**	2.60e-01	1.12	1.54	1.42		**	*
***MEF2D***	myocyte enhancer factor 2D	4209	4.21e-03	9.11e-02	**1.55e-03**	−1.84	1.24	−1.00	**		
***MEGF9***	multiple EGF-like-domains 9	1955	1.01e-04	**2.69e-04**	9.48e-01	1.62	1.76	1.69	*	**	*
***MERTK***	c-mer proto-oncogene tyrosine kinase	10461	6.83e-05	**2.96e-05**	6.07e-02	1.28	1.79	1.32	*	**	*
***MINPP1***	multiple inositol-polyphosphate phosphatase 1	9562	4.12e-02	**2.07e-03**	6.87e-01	1.51	1.54	1.28	*	*	
***MIR29A***	microRNA 29a	407021	6.91e-05	**9.06e-04**	3.57e-01	1.59	1.74	2.69			**
***MKI67***	antigen identified by monoclonal antibody Ki-67	4288	2.28e-02	6.57e-01	**1.27e-02**	2.18	−1.58	−1.13	**		
***MMP1***	matrix metallopeptidase 1 (interstitial collagenase)	4312	1.40e-04	**1.11e-03**	**3.61e-02**	−1.08	−1.44	−2.18		*	**
***MMP10***	matrix metallopeptidase 10 (stromelysin 2)	4319	5.96e-08	**1.29e-04**	**1.54e-03**	−1.02	−1.38	−2.37		*	**
***MMP3***	matrix metallopeptidase 3 (stromelysin 1, progelatinase)	4314	1.93e-05	**1.63e-03**	**2.51e-02**	−1.01	−1.63	−2.57	*	*	**
***MOB3C***	MOB kinase activator 3C	148932	2.40e-02	**1.54e-02**	**7.70e-03**	−1.78	1.01	−1.02	**		
***MYLIP***	myosin regulatory light chain interacting protein	29116	0.00e+00	**9.67e-07**	**8.89e-05**	2.84	1.52	1.12	**	**	
***NAV3***	neuron navigator 3	89795	7.05e-02	6.46e-01	**6.03e-03**	1.55	−1.42	−1.24	*	*	
***NCAPG***	non-SMC condensin I complex, subunit G	64151	8.61e-02	7.39e-01	**1.66e-02**	1.89	−1.41	−1.18	*		
***NEK2***	NIMA-related kinase 2	4751	5.47e-02	2.39e-01	**7.63e-03**	1.78	−1.23	−1.04	**		
***NINJ1***	ninjurin 1	4814	6.07e-02	**5.10e-03**	8.39e-02	−1.70	−1.25	−1.06	**		
***NIPAL4***	NIPA-like domain containing 4	348938	4.83e-03	**6.92e-04**	3.07e-01	−1.74	−1.31	−1.32	**		
***NR4A2***	nuclear receptor subfamily 4, group A, member 2	4929	2.09e-03	5.30e-01	**1.08e-03**	−1.85	1.21	1.31	**		
***NUAK2***	NUAK family, SNF1-like kinase, 2	81788	1.41e-02	**3.76e-04**	1.70e-01	−1.60	−1.19	−1.30	**		*
***NXF1***	nuclear RNA export factor 1	10482	1.95e-02	**4.17e-04**	7.68e-02	1.19	1.63	1.20		**	
***OLFM2***	olfactomedin 2	93145	9.51e-02	**4.42e-03**	7.98e-02	−1.65	−1.13	−1.14	**		
***OSBPL11***	oxysterol binding protein-like 11	114885	9.51e-02	**8.43e-03**	4.90e-01	1.84	1.30	1.37	*		
***OXTR***	oxytocin receptor	5021	1.11e-03	**9.51e-06**	**5.52e-03**	−1.10	−1.62	−1.32		**	**
***P4HA1***	prolyl 4-hydroxylase, alpha polypeptide I	5033	1.04e-04	4.09e-01	**3.61e-04**	1.58	−1.02	−1.89	**		**
***PCF11***	PCF11, cleavage and polyadenylation factor subunit, homolog (*S. cerevisiae*)	51585	2.42e-02	**3.67e-02**	**3.25e-02**	2.13	−1.01	1.06	**		
***PDGFB***	platelet-derived growth factor beta polypeptide	5155	2.00e-04	**6.09e-04**	9.34e-02	−2.09	−1.26	−1.36	**		
***PDK1***	pyruvate dehydrogenase kinase, isozyme 1	5163	1.91e-06	**2.69e-02**	**4.56e-04**	1.44	−1.19	−2.37	*		**
***PDZK1IP1***	PDZK1 interacting protein 1	10158	3.32e-02	**2.18e-03**	6.99e-02	−1.60	−1.03	−1.39	**		*
***PER1***	period circadian clock 1	5187	1.43e-02	6.69e-02	**3.66e-03**	−1.84	1.08	1.08	**		
***PFKFB4***	6-phosphofructo-2-kinase/fructose-2,6-biphosphatase 4	5210	0.00e+00	**3.32e-04**	**6.63e-05**	−1.06	1.04	−2.67			**
***PHLDA3***	pleckstrin homology-like domain, family A, member 3	23612	4.60e-02	**7.89e-04**	5.33e-01	−1.52	−1.27	−1.31	**		*
***PIK3R1***	phosphoinositide-3-kinase, regulatory subunit 1 (alpha)	5295	2.59e-03	**1.84e-02**	**2.19e-02**	2.40	1.14	−1.02	**		
***PIK3R3***	phosphoinositide-3-kinase, regulatory subunit 3 (gamma)	8503	1.86e-02	**4.86e-04**	5.59e-01	1.28	1.54	1.37		**	*
***PLAT***	plasminogen activator, tissue	5327	3.74e-02	**1.90e-02**	1.06e-01	−2.20	−1.18	−1.12	**		
***PLCB4***	phospholipase C, beta 4	5332	7.45e-06	**3.68e-02**	**2.37e-03**	1.73	−2.07	−2.14	*	**	**
***PLEKHG2***	pleckstrin homology domain containing, family G (with RhoGef domain) member 2	64857	3.71e-02	**3.43e-03**	7.01e-02	−1.72	−1.23	−1.08	**		
***PLEKHM1***	pleckstrin homology domain containing, family M (with RUN domain) member 1	9842	8.05e-02	9.03e-02	**1.43e-02**	−1.28	1.65	1.26		**	
***PLEKHM3***	pleckstrin homology domain containing, family M, member 3	389072	3.51e-02	**4.36e-03**	9.12e-01	1.57	1.78	1.56		*	
***PODXL***	podocalyxin-like	5420	1.55e-02	**8.88e-04**	**9.91e-03**	−1.05	−1.16	−1.69			**
***POLA1***	polymerase (DNA directed), alpha 1, catalytic subunit	5422	9.69e-02	3.40e-01	**2.19e-02**	1.95	−1.35	−1.00	**		
***PPP1R18***	protein phosphatase 1, regulatory subunit 18	170954	2.09e-02	**7.61e-03**	**1.02e-02**	−1.77	−1.06	−1.02	**		
***PPP1R3C***	protein phosphatase 1, regulatory subunit 3C	5507	1.19e-02	**9.34e-03**	7.03e-02	1.04	−1.54	−1.94		*	**
***PRDM1***	PR domain containing 1, with ZNF domain	639	0.00e+00	**1.29e-05**	**3.46e-06**	−2.47	−1.04	−1.01	**		
***PRKCA***	protein kinase C, alpha	5578	3.16e-03	**6.04e-04**	9.57e-01	1.48	1.59	1.54	*	*	*
***PRSS22***	protease, serine, 22	64063	2.23e-02	**3.34e-04**	8.39e-01	−1.41	−1.43	−1.31	**	**	
***PTCH1***	patched 1	5727	8.87e-02	**1.19e-02**	**6.15e-03**	−1.02	1.65	1.04		**	
***PTP4A3***	protein tyrosine phosphatase type IVA, member 3	11156	1.22e-04	**2.90e-04**	3.74e-01	−1.51	−1.95	−1.41	*	**	
***PXDC1***	PX domain containing 1	221749	3.90e-02	3.71e-01	**2.02e-03**	−1.66	1.16	1.19	**		
***RAI14***	retinoic acid induced 14	26064	9.76e-02	7.26e-01	**1.68e-02**	1.87	−1.31	−1.26	*		
***RASD2***	RASD family, member 2	23551	6.21e-02	**7.34e-04**	**2.19e-02**	−1.58	−1.08	−1.15	**		
***RASSF5***	Ras association (RalGDS/AF-6) domain family member 5	83593	9.60e-02	**2.47e-04**	6.95e-01	−1.29	−1.39	−1.25		**	
***RIPK4***	receptor-interacting serine–threonine kinase 4	54101	1.33e-03	**3.07e-04**	**1.37e-02**	−1.79	−1.10	−1.23	**		
***RND1***	Rho family GTPase 1	27289	9.23e-03	**1.56e-02**	5.23e-02	−1.12	1.59	2.06			**
***RNF103***	ring finger protein 103	7844	6.68e-02	**2.52e-03**	9.20e-01	1.44	1.51	1.37	*	*	
***RRM2***	ribonucleotide reductase M2	6241	5.93e-02	**3.12e-03**	**1.19e-02**	1.59	1.22	−1.04	**		
***S100A3***	S100 calcium binding protein A3	6274	0.00e+00	**7.47e-06**	5.36e-02	−2.37	−1.37	−2.05	**		**
***SAMD4A***	sterile alpha motif domain containing 4A	23034	7.19e-04	**4.28e-06**	7.70e-02	1.22	1.56	1.36	*	**	**
***SELE***	selectin E	6401	2.45e-03	**2.50e-03**	**1.08e-02**	−1.01	1.24	1.89			**
***SEMA4C***	sema domain, immunoglobulin domain (Ig), transmembrane domain (TM) and short cytoplasmic domain, (semaphorin) 4C	54910	9.90e-02	**1.27e-03**	8.89e-02	−1.53	−1.26	−1.08	**	*	
***SERPINA3***	serpin peptidase inhibitor, clade A (alpha-1 antiproteinase, antitrypsin), member 3	12	2.93e-03	**4.08e-03**	1.68e-01	−2.38	−1.29	−1.37	**		
***SERPINB9***	serpin peptidase inhibitor, clade B (ovalbumin), member 9	5272	4.70e-02	**4.96e-03**	1.48e-01	−1.07	−1.39	−1.72			**
***SERPINH1***	serpin peptidase inhibitor, clade H (heat shock protein 47), member 1, (collagen binding protein 1)	871	6.23e-02	**1.47e-03**	4.28e-01	−1.27	−1.27	−1.58			**
***SERPINI1***	serpin peptidase inhibitor, clade I (neuroserpin), member 1	5274	2.82e-02	**1.25e-03**	6.61e-01	1.54	1.28	1.46	*		*
***SLC2A1***	solute carrier family 2 (facilitated glucose transporter), member 1	6513	2.93e-03	**1.28e-03**	6.12e-02	−1.23	−1.19	−1.91			**
***SLC33A1***	solute carrier family 33 (acetyl-CoA transporter), member 1	9197	6.24e-02	**6.09e-04**	3.29e-01	1.42	1.42	1.16	**	**	
***SLC35D1***	solute carrier family 35 (UDP-glucuronic acid/UDP-N-acetylgalactosamine dual transporter), member D1	23169	5.29e-02	**3.06e-03**	6.97e-01	1.65	1.38	1.35	*		
***SLC6A14***	solute carrier family 6 (amino acid transporter), member 14	11254	8.97e-02	**5.67e-03**	8.88e-02	1.01	−1.46	−1.50		*	*
***SLC6A8***	solute carrier family 6 (neurotransmitter transporter, creatine), member 8	6535	2.16e-03	**1.14e-03**	**1.69e-02**	−1.03	1.70	1.47		**	**
***SMIM14***	small integral membrane protein 14	201895	7.25e-02	**3.85e-03**	4.08e-01	1.19	1.64	1.41		*	
***SNHG12***	small nucleolar RNA host gene 12 (non-protein coding)	85028	2.55e-04	**1.61e-05**	6.39e-02	1.66	1.38	1.21	**	**	*
***SNORA61***	small nucleolar RNA, H/ACA box 61	677838	7.99e-04	**6.54e-05**	7.40e-02	1.62	1.45	1.15	**	**	
***SNORD105***	small nucleolar RNA, C/D box 105	692229	1.18e-02	**5.73e-03**	9.15e-02	−1.82	−1.58	−1.00	**	*	
***SNORD13P2***	small nucleolar RNA, C/D box 13 pseudogene 2	6077	1.60e-02	8.33e-02	**5.74e-03**	−1.88	1.16	1.01	**		
***SNORD14E***	small nucleolar RNA, C/D box 14E	85391	1.44e-02	**1.01e-02**	9.15e-02	2.17	1.21	1.14	**		
***SNORD22***	small nucleolar RNA, C/D box 22	9304	1.49e-06	**3.21e-04**	**3.16e-02**	2.57	1.32	1.34	**		
***SNORD28***	small nucleolar RNA, C/D box 28	9300	4.12e-02	**1.90e-03**	1.47e-01	1.66	1.14	1.26	**		
***SNORD30***	small nucleolar RNA, C/D box 30	9299	1.45e-02	**3.29e-03**	**4.36e-02**	1.82	1.11	1.16	**		
***SNORD31***	small nucleolar RNA, C/D box 31	9298	5.13e-06	**9.26e-05**	**3.20e-03**	2.08	1.18	1.17	**		
***SNORD32A***	small nucleolar RNA, C/D box 32A	26819	6.96e-02	**1.74e-02**	**3.10e-02**	−1.66	−1.32	1.11	**		
***SPRY4***	sprouty homolog 4 (Drosophila)	81848	8.04e-02	**2.15e-03**	1.03e-01	−1.59	−1.09	−1.25	**		
***SRGN***	serglycin	5552	1.96e-02	**1.14e-04**	7.38e-02	−1.15	−1.28	−1.55		*	**
***STAT5A***	signal transducer and activator of transcription 5A	6776	8.68e-02	**6.33e-04**	6.45e-01	−1.22	−1.38	−1.40			**
***STC1***	stanniocalcin 1	6781	8.00e-04	**1.62e-03**	**1.37e-02**	−1.21	−1.06	−2.01			**
***STC2***	stanniocalcin 2	8614	9.30e-04	**1.85e-03**	**5.42e-03**	1.01	−1.19	−1.93			**
***STK17B***	serine/threonine kinase 17b	9262	1.23e-03	**1.93e-03**	1.18e-01	2.15	1.36	1.23	**		
***STX4***	syntaxin 4	6810	2.28e-03	**1.23e-04**	9.23e-01	−1.49	−1.40	−1.43	**	*	**
***SYDE1***	synapse defective 1, Rho GTPase, homolog 1 (*C. elegans*)	85360	9.00e-02	**9.80e-04**	4.81e-01	−1.43	−1.19	−1.40	**		*
***TAGLN***	transgelin	6876	3.45e-03	**3.65e-04**	6.89e-01	−1.43	−1.37	−1.61	*	*	**
***TAS2R4***	taste receptor, type 2, member 4	50832	9.62e-02	**1.10e-02**	1.86e-01	1.83	1.15	1.22	**		
***TFRC***	transferrin receptor (p90, CD71)	7037	7.32e-02	3.99e-01	**2.32e-02**	2.18	−1.21	−1.23	**		
***TGFB1I1***	transforming growth factor beta 1 induced transcript 1	7041	8.92e-05	**2.58e-05**	9.24e-01	−1.47	−1.48	−1.55	**	**	**
***TGFB2***	transforming growth factor, beta 2	7042	1.50e-05	**2.51e-03**	**3.22e-03**	1.19	−2.14	−1.52		**	*
***TICAM1***	toll-like receptor adaptor molecule 1	148022	4.13e-03	**8.38e-03**	**8.32e-04**	−1.79	1.05	1.01	**		
***TLE1***	transducin-like enhancer of split 1 (E(sp1) homolog, Drosophila)	7088	3.37e-03	**1.25e-02**	**3.72e-04**	−1.23	1.70	1.14	*	**	
***TM4SF18***	transmembrane 4L six family member 18	116441	3.05e-02	6.72e-02	**2.10e-03**	1.31	−1.24	−1.55	*		**
***TMEM2***	transmembrane protein 2	23670	2.67e-02	**5.08e-03**	6.27e-01	2.00	1.44	1.53	*		
***TNFAIP6***	tumor necrosis factor, alpha-induced protein 6	7130	1.27e-02	**4.06e-03**	2.87e-01	−1.16	−1.70	−1.84		*	*
***TNFRSF11B***	tumor necrosis factor receptor superfamily, member 11b	4982	6.74e-06	**4.09e-04**	2.27e-01	−1.49	−2.62	−1.70		**	*
***TNFSF18***	tumor necrosis factor (ligand) superfamily, member 18	8995	0.00e+00	**3.06e-06**	3.10e-01	−2.41	−4.24	−3.41	**	**	**
***TOP2A***	topoisomerase (DNA) II alpha 170 kDa	7153	4.62e-02	8.29e-01	**1.18e-02**	1.90	−1.42	−1.24	**		
***TRAF1***	TNF receptor-associated factor 1	7185	2.49e-04	**8.97e-05**	2.32e-01	−1.69	−1.50	−1.26	**	**	
***TRIP6***	thyroid hormone receptor interactor 6	7205	8.37e-02	**9.63e-04**	6.24e-01	−1.48	−1.30	−1.26	**	*	
***TSGA10***	testis specific, 10	80705	6.19e-02	**6.67e-03**	**1.65e-02**	1.68	1.07	1.04	**		
***TTLL3***	tubulin tyrosine ligase-like family, member 3	26140	3.38e-03	**1.07e-03**	**1.29e-02**	−1.04	1.65	1.45		**	**
***UBALD1***	UBA-like domain containing 1	124402	1.29e-02	9.81e-02	**5.00e-03**	−1.88	1.23	−1.03	**		
***ULBP3***	UL16 binding protein 3	79465	5.51e-02	**3.20e-04**	1.21e-01	1.30	1.50	1.12	*	**	
***ULK1***	unc-51-like kinase 1 (*C. elegans)*	8408	8.48e-02	4.11e-01	**2.45e-03**	−1.44	1.33	1.27	**	*	
***USP38***	ubiquitin specific peptidase 38	84640	8.54e-03	**1.46e-04**	4.15e-01	1.53	1.34	1.27	**	*	*
***VAMP1***	vesicle-associated membrane protein 1 (synaptobrevin 1)	6843	1.08e-03	**3.77e-05**	4.65e-01	1.49	1.48	1.28	**	**	*
***VLDLR***	very low density lipoprotein receptor	7436	4.04e-02	**4.49e-04**	**2.18e-03**	−1.05	−1.08	−1.61			**
***ZC3H7B***	zinc finger CCCH-type containing 7B	23264	6.99e-02	**4.01e-04**	8.97e-01	−1.39	−1.30	−1.32	**		
***ZC3HAV1***	zinc finger CCCH-type, antiviral 1	56829	4.54e-02	**3.89e-03**	2.25e-01	1.74	1.29	1.17	**		
***ZCCHC14***	zinc finger, CCHC domain containing 14	23174	3.05e-02	**1.36e-03**	9.05e-02	1.11	1.66	1.27		**	
***ZNF253***	zinc finger protein 253	56242	5.52e-02	**2.43e-02**	1.07e-01	2.18	1.16	1.11	**		
***ZNF426***	zinc finger protein 426	79088	6.98e-02	**3.42e-03**	7.62e-01	1.55	1.51	1.30	*	*	
***ZNF48***	zinc finger protein 48	197407	5.05e-02	**5.04e-03**	**1.14e-02**	−1.67	−1.07	−1.03	**		
***ZNF487P***	zinc finger protein 487, pseudogene	642819	5.33e-05	**1.01e-03**	5.62e-02	1.47	2.43	1.18		**	
***ZNF724P***	zinc finger protein 724, pseudogene	440519	3.69e-02	1.45e-01	**1.73e-02**	2.07	−1.05	−1.15	**		
***ZNF737***	zinc finger protein 737	100129842	2.08e-02	**6.03e-03**	3.48e-01	2.11	1.29	1.46	**		
***ZSWIM6***	zinc finger, SWIM-type containing 6	57688	4.63e-02	**5.34e-03**	4.44e-01	1.55	1.78	1.22		*	

*P*-values in bold if significant at two-factor ANOVA (<0.05).

FDR: false discovery rate, according to BETR analysis. *P*_*treatment*_: *P*-value for treatment effect; and *P*_*interaction*_: *P*-value for interaction effect, at two-factor ANOVA. FC: fold-change in Aminaphtone-treated *vs.* untreated IL-1β-stimulated endothelial cells. AMNA: Aminaphtone. Pairwise: pairwise significant at two-tailed *t*-test.

* *P*<0.05.

** *P*<0.01 at two-tailed *t*-test.

**Table 2:** Gene sets significantly modulated by Aminaphtone in treated *vs.* untreated IL-1β-stimulated endothelial cells.

**GO Category**^a^	**GO Ontology**	**GO term**	**#genes**	**LS**	**KS**	**Gene list**
0005975	BP	carbohydrate metabolic process	11	0.0805	**0.0028**	*DDIT4*, *ENO2*, *FUT11*, *GCLC*, *HK2*, *HMMR*, *PDGFB*, *PDK1*, *PFKFB4*, *PPP1R3C*, *SLC2A1*
0007166	BP	cell surface receptor signaling pathway	46	0.0260	**0.0020**	*AAK1*, *BCL2L11*, *BMP6*, *CBLB*, *CITED2*, *CX3CL1*, *DDIT4*, *DDR1*, *DKK1*, *EBI3*, *ECT2*, *EDN1*, *EFNA1*, *EFNB2*, *EIF4B*, *EIF4EBP2*, *FOS*, *FST*, *GAREM*, *GPR56*, *GRB7*, *IL1B*, *IL7R*, *KCTD11*, *LIF*, *MERTK*, *OXTR*, *PDGFB*, *PIK3R1*, *PIK3R3*, *PLAT*, *PLEKHG2*, *PRDM1*, *PRKCA*, *PTCH1*, *SEMA4C*, *STAT5A*, *STC1*, *STC2*, *TGFB1I1*, *TGFB2*, *TICAM1*, *TLE1*, *TNFSF18*, *ULK1*, *VLDLR*
0022411	BP	cellular component disassembly	7	**0.0042**	0.8859	*BNIP3*, *DDIT4*, *DDR1*, *MMP1*, *MMP10*, *MMP3*, *TOP2A*
0048732	BP	gland development	11	**0.0031**	**0.0022**	*BCL2L11*, *CITED2*, *DDR1*, *ELF3*, *HK2*, *ID2*, *OXTR*, *PDGFB*, *PTCH1*, *STAT5A*, *TGFB2*
0042592	BP	homeostatic process	26	**0.0015**	0.2886	*BCL2L11*, *BNIP3*, *CITED2*, *CTSK*, *EDN1*, *EGR2*, *GCLC*, *GCLM*, *GPR89A*, *HK2*, *HMOX1*, *ID2*, *IL1B*, *IL7R*, *MYLIP*, *OXTR*, *POLA1*, *PRKCA*, *PTCH1*, *SERPINA3*, *STAT5A*, *STC1*, *STC2*, *TFRC*, *TGFB2*, *TNFRSF11B*
0002376	BP	immune system process	47	**0.0015**	0.0284	*ANXA3*, *BCL2L11*, *BCL3*, *BMP6*, *BNIP3*, *CBLB*, *CCL22*, *CD70*, *CD83*, *CITED2*, *CLEC4E*, *CSF2*, *CTSK*, *CX3CL1*, *CXCL3*, *CXCL6*, *DDIT4*, *EBI3*, *EDN1*, *FOS*, *FST*, *GRB7*, *HMOX1*, *ID2*, *IL1B*, *IL32*, *IL7R*, *KIF23*, *LIF*, *MARCH8*, *MERTK*, *MMP1*, *PDGFB*, *PIK3R1*, *PIK3R3*, *PODXL*, *PRDM1*, *PRKCA*, *SELE*, *SERPINB9*, *STAT5A*, *TFRC*, *TGFB2*, *TICAM1*, *TNFSF18*, *ULBP3*, *ZC3HAV1*
0006954	BP	inflammatory response	15	0.0131	**0.0047**	*BMP6*, *CCL22*, *CX3CL1*, *CXCL3*, *CXCL6*, *ELF3*, *FOS*, *HMOX1*, *IL1B*, *PRKCA*, *SELE*, *SERPINA3*, *STAT5A*, *TICAM1*, *TNFAIP6*
0043066	BP	negative regulation of apoptotic process	20	**0.0028**	0.0677	*AGAP2*, *BCL3*, *BNIP3*, *CITED2*, *CSF2*, *CTH*, *CX3CL1*, *EDN1*, *GCLC*, *GCLM*, *HMOX1*, *IL1B*, *NR4A2*, *NUAK2*, *PIK3R1*, *PRKCA*, *SERPINB9*, *STAT5A*, *TLE1*, *TNFSF18*
0007389	BP	pattern specification process	6	0.0137	**0.0006**	*CITED2*, *DKK1*, *EDN1*, *EGR2*, *FST*, *PTCH1*
0010646	BP	regulation of cell communication	49	0.1744	**0.0034**	*AAK1*, *ACAP1*, *AGAP2*, *ANKRD1*, *ARHGEF26*, *BCL2L11*, *BCL3*, *BMP6*, *BNIP3*, *CBLB*, *CITED2*, *CSF2*, *CTH*, *DDIT4*, *DKK1*, *DUSP8*, *ECT2*, *EDN1*, *EFNA1*, *EGR2*, *FST*, *GAREM*, *GPR89A*, *HMOX1*, *IL1B*, *KCTD11*, *LIF*, *LMCD1*, *OXTR*, *PDGFB*, *PHLDA3*, *PLAT*, *PLEKHG2*, *PRDM1*, *PRKCA*, *PTCH1*, *RASD2*, *SEMA4C*, *SLC2A1*, *SPRY4*, *SYDE1*, *TGFB1I1*, *TGFB2*, *TICAM1*, *TLE1*, *TRAF1*, *TRIP6*, *ULK1*, *ZC3HAV1*
0042127	BP	regulation of cell proliferation	23	**0.0019**	0.0322	*BMP6*, *BRCA1*, *CBLB*, *CSF2*, *DDR1*, *EBI3*, *EDN1*, *GAREM*, *HILPDA*, *HMOX1*, *ID2*, *IL1B*, *KCTD11*, *LIF*, *PDGFB*, *PRDM1*, *PRKCA*, *PTCH1*, *STAT5A*, *TGFB1I1*, *TGFB2*, *TICAM1*, *TNFSF18*
0045619	BP	regulation of lymphocyte differentiation	5	**0.0009**	**0.0034**	*CD83*, *ID2*, *IL7R*, *PRDM1*, *STAT5A*
0051246	BP	regulation of protein metabolic process	38	0.0567	**0.0032**	*AGAP2*, *BCL3*, *BMP6*, *BRCA1*, *BUB1B*, *CBLB*, *CSF2*, *DDIT4*, *DKK1*, *DUSP8*, *EBI3*, *ECT2*, *EDN1*, *EFNA1*, *EIF4B*, *EIF4EBP2*, *GCLC*, *GRB7*, *HMOX1*, *IL1B*, *KRT17*, *LIF*, *LPIN1*, *MYLIP*, *PDGFB*, *PLAT*, *PRKCA*, *RASD2*, *SAMD4A*, *SERPINA3*, *SERPINB9*, *SERPINH1*, *SERPINI1*, *SPRY4*, *STAT5A*, *TGFB2*, *TICAM1*, *VLDLR*
0051090	BP	regulation of sequence-specific DNA binding transcription factor activity	10	**0.0020**	0.0058	*ANXA3*, *CTH*, *FOS*, *HMOX1*, *ID2*, *IL1B*, *PTCH1*, *TICAM1*, *TNFSF18*, *TRAF1*
0006357	BP	regulation of transcription from RNA polymerase II promoter	30	**0.0035**	0.6000	*ANKRD1*, *BCL3*, *BMP6*, *BRCA1*, *CITED2*, *DKK1*, *EDN1*, *EFNA1*, *EGR2*, *ELF3*, *ERF*, *FOS*, *FST*, *HMOX1*, *ID2*, *IL1B*, *LIF*, *LMCD1*, *LPIN1*, *MEF2D*, *NR4A2*, *PER1*, *PIK3R1*, *POLA1*, *PRDM1*, *PTCH1*, *RRM2*, *STAT5A*, *TOP2A*, *VLDLR*
0036293	BP	response to decreased oxygen levels	14	**0.0024**	**0.0002**	*ANKRD1*, *BNIP3*, *CITED2*, *DDIT4*, *EDN1*, *HMOX1*, *NR4A2*, *OXTR*, *PDGFB*, *PDK1*, *PLAT*, *STC1*, *STC2*, *TGFB2*
0009605	BP	response to external stimulus	32	**0.0001**	0.0071	*AGAP2*, *ANKRD1*, *BNIP3*, *CCL22*, *CITED2*, *CX3CL1*, *CXCL3*, *CXCL6*, *CYP24A1*, *EDN1*, *EFNB2*, *EGR2*, *ELOVL4*, *FOS*, *HMOX1*, *ID2*, *IL1B*, *NR4A2*, *NUAK2*, *PDGFB*, *PER1*, *PLAT*, *PRKCA*, *RND1*, *SELE*, *SLC2A1*, *STAT5A*, *STC1*, *STC2*, *TGFB2*, *TNFRSF11B*, *ULK1*
0010033	BP	response to organic substance	48	**<0.0001**	0.0168	*ANKRD1*, *BMP6*, *BRCA1*, *CD83*, *CITED2*, *CSF2*, *CTH*, *CX3CL1*, *CYP24A1*, *DKK1*, *DNAJB1*, *EBI3*, *EDN1*, *EGR2*, *EIF4B*, *EIF4EBP2*, *FOS*, *GAREM*, *GCLC*, *HCAR1*, *HMOX1*, *IDI1*, *IL1B*, *IL7R*, *LOX*, *LPIN1*, *MMP3*, *NR4A2*, *OXTR*, *PDGFB*, *PIK3R1*, *PIK3R3*, *PLAT*, *PRDM1*, *PRKCA*, *PTCH1*, *SELE*, *SERPINB9*, *SERPINH1*, *STAT5A*, *STC1*, *STC2*, *TGFB1I1*, *TGFB2*, *TICAM1*, *TNFRSF11B*, *TNFSF18*, *ZC3HAV1*
0048511	BP	rhythmic process	6	**0.0033**	0.0194	*EGR2*, *ID2*, *OXTR*, *PER1*, *STAT5A*, *TGFB2*
0046903	BP	secretion	20	0.0238	**0.0008**	*ANKRD1*, *ANXA3*, *BMP6*, *CLEC4E*, *DDR1*, *EDN1*, *FST*, *HK2*, *HMOX1*, *IL1B*, *LIF*, *MERTK*, *OXTR*, *PDGFB*, *PRKCA*, *SLC2A1*, *SRGN*, *STAT5A*, *TGFB2*, *VAMP1*
0005125	MF	cytokine activity	15	0.0061	**0.0047**	*BMP6*, *CCL22*, *CD70*, *CSF2*, *CX3CL1*, *CXCL3*, *CXCL6*, *EBI3*, *EDN1*, *IL1B*, *IL32*, *LIF*, *TGFB2*, *TNFRSF11B*, *TNFSF18*
0004175	MF	endopeptidase activity	6	**0.0004**	0.1037	*CTSK*, *MMP1*, *MMP10*, *MMP3*, *PLAT*, *PRSS22*
0001071	MF	nucleic acid binding transcription factor activity	11	**0.0006**	0.0382	*BCL3*, *CEBPD*, *CITED2*, *EGR2*, *ELF3*, *ERF*, *FOS*, *MEF2D*, *NR4A2*, *PRDM1*, *STAT5A*
0000988	MF	protein binding transcription factor activity	11	0.0990	**0.0032**	*ANKRD1*, *BRCA1*, *CITED2*, *ELF3*, *ERF*, *LIF*, *LMCD1*, *LPIN1*, *PER1*, *TGFB1I1*, *TLE1*
0019904	MF	protein domain specific binding	5	**0.0031**	0.0167	*ACAP1*, *CITED2*, *EGR2*, *IL1B*, *KHDRBS3*
0001067	MF	regulatory region nucleic acid binding	6	**0.0040**	0.0378	*BRCA1*, *EGR2*, *FOS*, *MEF2D*, *PER1*, *STAT5A*
0031012	CC	extracellular matrix	9	**0.0034**	**0.0040**	*LAMB3*, *LMCD1*, *LOX*, *MMP1*, *MMP10*, *MMP3*, *TGFB1I1*, *TGFB2*, *TNFRSF11B*
0005615	CC	extracellular space	31	**0.0003**	**<0.0001**	*BMP6*, *CCL22*, *CD70*, *CSF2*, *CTSK*, *CX3CL1*, *CXCL3*, *CXCL6*, *DKK1*, *EBI3*, *EDN1*, *HILPDA*, *HMOX1*, *IGFL1*, *IL1B*, *IL32*, *LIF*, *LMCD1*, *LOX*, *MERTK*, *MMP10*, *MMP3*, *PLAT*, *SELE*, *SERPINB9*, *SRGN*, *STC1*, *TGFB2*, *TNFRSF11B*, *TNFSF18*, *VLDLR*


**KEGG Pathway**^b^	**Pathway description**	**#genes**	**LS**	**KS**	**Gene list**
hsa04060	Cytokine-cytokine receptor interaction	13	0.0287	0.0095	*CCL22*, *CD70*, *CSF2*, *CX3CL1*, *CXCL3*, *CXCL6*, *IL1B*, *IL7R*, *LIF*, *PDGFB*, *TGFB2*, *TNFRSF11B*, *TNFSF18*


**Broad MSigDB Curated Gene Set**^c^	**#genes**	**LS**	**KS**	**Gene list**
HINATA_NFKB_TARGETS_KERATINOCYTE_UP	13	**0.0004**	0.0095	*CD83*, *CSF2*, *CXCL3*, *CXCL6*, *EFNA1*, *IL1B*, *IL32*, *IL7R*, *MMP1*, *PLAT*, *STAT5A*, *TNFAIP6*, *TRAF1*
HINATA_NFKB_TARGETS_FIBROBLAST_UP	6	**0.0027**	0.1123	*CD83*, *CXCL3*, *CXCL6*, *IL1B*, *MMP1*, *SELE*
SCHOEN_NFKB_SIGNALING	5	0.0192	**0.0036**	*CSF2*, *EDN1*, *IL1B*, *NUAK2*, *SERPINA3*
ELVIDGE_HYPOXIA_UP	19	**0.0017**	**0.0010**	*AK4*, *BNIP3*, *CITED2*, *DDIT4*, *DDR1*, *ELF3*, *ENO2*, *FOS*, *HK2*, *LOX*, *P4HA1*, *PDGFB*, *PDK1*, *PRKCA*, *SAMD4A*, *SLC2A1*, *STC1*, *STC2*, *VLDLR*
ELVIDGE_HIF1A_AND_HIF2A_TARGETS_DN	14	**0.0036**	**0.0001**	*AK4*, *BNIP3*, *CITED2*, *ENO2*, *FOS*, *HK2*, *LOX*, *P4HA1*, *PDGFB*, *PDK1*, *SAMD4A*, *SLC2A1*, *STC1*, *VLDLR*
ELVIDGE_HYPOXIA_BY_DMOG_UP	17	**0.0038**	**0.0023**	*AK4*, *BNIP3*, *CITED2*, *DDIT4*, *ELF3*, *ENO2*, *FOS*, *HK2*, *LOX*, *P4HA1*, *PDK1*, *PRKCA*, *SAMD4A*, *SLC2A1*, *STC1*, *STC2*, *VLDLR*
ELVIDGE_HIF1A_TARGETS_DN	13	0.0114	**0.0002**	*AK4*, *BNIP3*, *CITED2*, *ENO2*, *FOS*, *HK2*, *LOX*, *P4HA1*, *PDGFB*, *PDK1*, *SLC2A1*, *STC1*, *VLDLR*
WINTER_HYPOXIA_METAGENE	18	**0.0022**	0.0376	*BNIP3*, *CITED2*, *DDIT4*, *EDN1*, *EFNA1*, *ELF3*, *FOS*, *HK2*, *HMOX1*, *ID2*, *LOX*, *P4HA1*, *PDGFB*, *PFKFB4*, *SLC2A1*, *SLC6A8*, *STC2*, *TFRC*
PID_HIF1_TFPATHWAY	9	**0.0022**	0.2078	*BNIP3*, *CITED2*, *EDN1*, *FOS*, *HK2*, *HMOX1*, *ID2*, *SLC2A1*, *TFRC*
MENSE_HYPOXIA_UP	10	**0.0028**	**0.0004**	*BNIP3*, *CEBPD*, *ENO2*, *HK2*, *LOX*, *P4HA1*, *PDK1*, *PFKFB4*, *PPP1R3C*, *STC2*
LEONARD_HYPOXIA	11	0.0061	**0.0007**	*AK4*, *BNIP3*, *DDIT4*, *EFNA1*, *HK2*, *P4HA1*, *PDGFB*, *PFKFB4*, *PPP1R3C*, *SLC2A1*, *STC2*
JIANG_HYPOXIA_NORMAL	15	0.0134	**0.0009**	*AK4*, *BNIP3*, *CITED2*, *CNNM4*, *EIF4B*, *ENO2*, *HMOX1*, *KHDRBS3*, *LOX*, *P4HA1*, *PFKFB4*, *PPP1R3C*, *RIPK4*, *SLC2A1*, *STC2*
KRIEG_HYPOXIA_VIA_KDM3A	5	0.0182	**0.0026**	*C15orf48*, *CLEC4E*, *EDN1*, *HMOX1*, *LAMB3*
FARDIN_HYPOXIA_11	6	0.0200	**0.0029**	*AK4*, *BNIP3*, *DDIT4*, *FUT11*, *PDK1*, *PFKFB4*
NAGASHIMA_EGF_SIGNALING_UP	8	**0.0008**	**0.0007**	*AREG*, *DNAJB1*, *EDN1*, *EGR2*, *FOS*, *LIF*, *NR4A2*, *TNFRSF11B*
ZHANG_RESPONSE_TO_IKK_INHIBITOR_AND_TNF_UP	22	**0.0021**	0.0070	*BCL3*, *C15orf48*, *CD83*, *CXCL3*, *DDIT4*, *EDN1*, *EFNA1*, *GCLC*, *IGFL1*, *IL1B*, *IL32*, *IL7R*, *LAMB3*, *LIF*, *MMP10*, *NAV3*, *NINJ1*, *SAMD4A*, *SEMA4C*, *SERPINB9*, *TNFAIP6*, *TRAF1*
BASSO_CD40_SIGNALING_UP	8	**0.00498**	0.0093	*CCL22*, *CD83*, *DDIT4*, *IL1B*, *PTP4A3*, *SRGN*, *STAT5A*, *TRAF1*
WIERENGA_STAT5A_TARGETS_GROUP2	5	0.0163	**0.0036**	*CCL22*, *CD83*, *CSF2*, *IL7R*, *TRAF1*

GO ontology: BP=biological process, MF=molecular function, and CC=cellular component.

a GO (Gene Ontology) categories,

b KEGG (Kyoto Encyclopedia of Genes and Genomes) pathways, and

c MsigDB (Broad Institute Molecular Signature Database) curated gene sets found to be significant at the 0.005 significance level of either the LS or KS permutation tests. For each Gene Set, the table lists the unique identifier, the number of genes differentially modulated, the Fisher (LS) and Kolmogorov–Smirnov (KS) permutation *P*-values (*P*-values <0.005 are in bold), and the list of modulated genes.
