# Supplementary material for: Time-course gene expression data on the transcriptional effects of Aminaphtone on ECV304 endothelial cells
Source: Data Brief. 2016 Jul 2;8:836–50. doi: 10.1016/j.dib.2016.06.051 (PMC4957571; doi:10.1016/j.dib.2016.06.051)
Supplement: Supplementary file 1 — Supplementary material [file mmc1.docx]

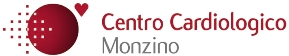


Gualtiero I. Colombo, MD, PhD

Head, Lab. of Immunology and Functional Genomics

Centro Cardiologico Monzino IRCCS

Via Parea, 4 – 20138 Milano, Italy

**Disclosure and Conflicts of Interest**

6/19/2016

Manuscript title: "***Time-course gene expression data on the transcriptional effects of Aminaphtone on ECV304 endothelial cells***".

Conflicts of interest: none.


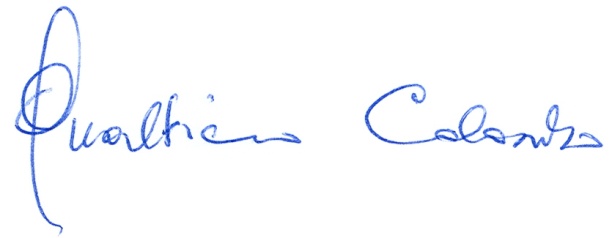


Gualtiero I. Colombo, MD, PhD

Head, Lab. of Immunology and Functional Genomics
